# The impact of indigenous cultural identity and cultural engagement on violent offending

**DOI:** 10.1186/s12889-017-4603-2

**Published:** 2017-07-24

**Authors:** Stephane M. Shepherd, Rosa Hazel Delgado, Juanita Sherwood, Yin Paradies

**Affiliations:** 1 Centre for Forensic Behavioural Science, Victorian Institute of Forensic Mental Health, Swinburne University of Technology, Melbourne, Australia; 2 Department of Psychology, University of Nebraska-Lincoln, Lincoln, USA; 3 National Centre for Cultural Competence, University of Sydney, Sydney, Australia; 4 Alfred Deakin Research Institute for Citizenship and Globalisation, Deakin University, Melbourne, Australia

**Keywords:** Correctional health care, Minority health, Indigenous prisoners, Cultural identity, Violence

## Abstract

**Background:**

Possessing a strong cultural identity has been shown to protect against mental health symptoms and buffer distress prompted by discrimination. However, no research to date has explored the protective influences of cultural identity and cultural engagement on violent offending. This paper investigates the relationships between cultural identity/engagement and violent recidivism for a cohort of Australian Indigenous people in custody.

**Methods:**

A total of 122 adults from 11 prisons in the state of Victoria completed a semi-structured interview comprising cultural identification and cultural engagement material in custody. All official police charges for violent offences were obtained for participants who were released from custody into the community over a period of 2 years.

**Results:**

No meaningful relationship between cultural identity and violent recidivism was identified. However a significant association between cultural engagement and violent recidivism was obtained. Further analyses demonstrated that this relationship was significant only for participants with a strong Indigenous cultural identity. Participants with higher levels of cultural engagement took longer to violently re-offend although this association did not reach significance.

**Conclusions:**

For Australian Indigenous people in custody, ‘cultural engagement’ was significantly associated with non-recidivism. The observed protective impact of cultural engagement is a novel finding in a correctional context. Whereas identity alone did not buffer recidivism directly, it may have had an indirect influence given its relationship with cultural engagement. The findings of the study emphasize the importance of culture for Indigenous people in custody and a greater need for correctional institutions to accommodate Indigenous cultural considerations.

## Background

A positive cultural identity can provide an individual with a sense of belonging, purpose, social support & self-worth [1]. This process may occur through an attachment to a cultural group whereby belief systems, values, obligations and practices are shared and reinforced by in-group members [2]. The potential health benefits of sustaining a strong cultural identity and/or participating in cultural activities have been documented in prior research with Indigenous and other non-white samples in Western settings. For example, possessing a strong cultural identity has been found to promote resilience, enhance self-esteem, engender pro-social coping styles and has served as a protective mechanism against mental health symptoms [3–9]. Moreover, cultural identity may buffer discrimination-induced distress [10–15].

Sustaining a strong cultural identity is a key component of Social and Emotional Wellbeing, an Indigenous Australian framework of health [16]. Here, an identity can be cultivated and maintained through participating in cultural events and developing a connection to family, community and traditional lands [16]. The strengthening of culture serves to build resilience and positive coping mechanisms facilitating life balance and protecting against adverse life experiences including ‘the impact of history in trauma and loss’ [17]. This includes the fostering of effective responses to stigma, discrimination and the ongoing impacts of colonisation [17]. Identifying and engaging in Indigenous cultures has been linked with enhanced self-assessed health, improved educational and employment outcomes, and greater life satisfaction.

Whereas the protective qualities of cultural identity/attachment on socio-economic and health indicators are progressively theorized and explored, little attention has been afforded to the protective influence Indigenous Australian culture may have on law breaking activities and violence. Prior research has explored (and did not find evidence for) the notion that Indigenous culture may in fact prompt violent victimization [18]. Other Australian research found that cultural ‘strengths’ were associated with a reduction in the prevalence of arrests for Indigenous Australians in remote communities [19]. Both the above studies utilized data from the National Aboriginal and Torres Strait Islander Social Survey (NATSISS), a cross-sectional periodic survey on the socio-economic circumstances of Aboriginal and Torres Strait Islander Australians. It is clear that there is a dearth of regional evidence for the effects of culture on problem behaviours. As such, the protective qualities of Indigenous cultural identity and cultural engagement on future offending warrant further exploration. This is important in light of the increasing rates of Indigenous imprisonment in Australia [20] and recent efforts by state departments to acknowledge the importance of Indigenous culture in reducing contact with the justice system [21].

The forensic literature has underscored the influence of general protective factors such as involvement in pro-social activities and possessing positive attitudes on criminal desistance [22]. Client protective factors or ‘strengths’ are now a common part of forensic risk management frameworks and violence risk instruments [23]. However, little explicit attention is afforded to culture in such structures [24]. It is therefore of interest to determine whether cultural identity/attachment serves as a meaningful protective factor for Indigenous Australians which may help inform correctional therapeutic initiatives to reduce recidivism.

This study seeks to identity the relationship between cultural identity, cultural engagement and violent recidivism for a sample of Indigenous people in custody, the first of its kind internationally. In line with prior literature on identity, we partitioned notions of cultural identity and cultural engagement [1, 25, 26]. It is conceivable for an Indigenous person to possess a strong cultural identity yet have reduced means to: engage in cultural activities, express culturally typical behaviours, and/or establish a meaningful cultural connection. Others may prefer their cultural identity to be purely nominal while others still may have high levels of cultural engagement without an expressed and explicit cultural identity, or with an identity that is hidden or repressed. Cultural involvement beyond basic affiliation may also be constrained owing to the legacies of government sanctioned child removal and assimilationist policies in Australia. Moreover, Indigeneity has often been demarcated by the state – over 60 classifications since white settlement [27] - overlooking the heterogeneity of cultural practices among Aboriginal and Torres Strait Islander peoples. Furthermore, a chasm between cultural identity and cultural expression may be more likely to occur in prison settings where opportunities to access cultural resources may be limited.

With scant prior literature to guide our hypotheses, we cautiously anticipate that a strong cultural identity may induce a lower likelihood of violent re-offence. However, we expect this association to perhaps be stronger when coupled with greater cultural engagement in custody. We also predict that higher levels of cultural engagement in custody would increase the time before re-offence.

## Methods

### Participant details

Data were collected for 122 (Male = 107; Female = 15) remanded and sentenced individuals in custody in the state of Victoria, Australia. All clients were formally registered as Aboriginal and Torres Strait Islander persons with Victorian prison services.

The final sample comprised 119 participants (Male = 104: Female = 15). The capture rate was high over the course of 8 months enabling a representative sample - Aboriginal and Torres Strait Islander people are estimated to be only 0.9% of the Victorian general population [28]. The proportion of Aboriginal and Torres Strait Islander prisoners in Victoria is 8%, over 8-fold over-represented compared to the general population, but, nonetheless, the lowest of any state in Australia [29]. Three participants were excluded from the study due to incomplete cultural identity information.

The mean age of the sample was 34.28 (*SD* = 10.29, *range* = 19 - 63). Over half of the cohort (56.3%, *N* = 67) was born in Victoria, 19.3% (*N* = 23) were born in New South Wales and 9.2% (*N* = 11) were born in Queensland. The mean number of lifetime episodes in adult custody was 5.14 (*SD* = 5.46). The majority of the sample had been previously charged with a violent offence (60.5%, *N* = 72).

### Procedure

Data for this analysis was obtained from the Koori Prisoner Mental Health and Cognitive Function Study (KPMHS) [30] database. The KPMHS was conducted by the Centre for Forensic Behavioral Science under contract from the Victorian Department of Justice to investigate the mental health needs of Koori prisoners. Ethical approval to utilize the database for the purposes of this study was obtained from the Victorian Department of Justice Human Research Ethics Committee, the Koori Justice Unit and Swinburne University Human Research Ethics Committee.

Data collection took place from January 2012 until October 2012. All remanded and sentenced Aboriginal and Torres Strait Islander prisoners from 11 regional and metropolitan prisons Victoria-wide were approached to participate in the study. The distribution of participants by correctional centre ranged from 2.5% (*N* = 3) - 18% (*N* = 22). Participants from the KPMHS study were interviewed and administered an amalgamated health related questionnaire in custody. Information obtained included Social and Emotional Wellbeing factors (inclusive of cultural identification and cultural engagement material), mental health symptoms, service use access and cognitive functioning.

Aboriginal Wellbeing/Liaison Officers at each prison informed prospective participants about the study. Individuals who demonstrated an interest in participating in the study then met with the interviewers who explained the study to them in greater detail. Prior to the interview, an Aboriginal and Torres Strait Islander research officer verbally reviewed a study explanatory statement with the participant and provided an opportunity for the participant to ask questions. Participants who wished to take part were asked to sign a consent form acknowledging their understanding of the study. Interviews were conducted by two assessors, the Aboriginal and Torres Strait Islander research officer and a mental health clinician. All interviews were conducted in private rooms visible to prison staff. Participation in the study was voluntary and participants could choose not to answer any questions, or terminate the interview at any time, if preferred. The duration of interviews ranged from 50 to 240 min in length. For the purposes of this study, selected materials from the amalgamated questionnaire were chosen for examination. The full sample from the original KPMHS was included in the analysis.

Follow-up data were collected for participants who were released from custody into the community. Criminal histories from the Victoria Police Law Enforcement Assistance Program (LEAP) database were obtained for all consenting participants for 2 years post custodial interview. The LEAP database records all contacts people in Victoria have with the Victoria Police, both as offenders and victims. Violence Recidivism is defined as any police charge for a violent offence post assessment. Violent crimes are defined as acts intended to cause or threaten to cause physical harm.

### Measures

#### Cultural identity

Aboriginal and Torres Strait Islander identity was measured via an abbreviated version of the Aboriginal and Torres Strait Islander Identity Scale (ATIS). The seven items that comprise the ATIS were selected from a broader Social and Emotional Wellbeing and needs/service access questionnaire created by an advisory group of Australian Aboriginal psychologists for the KPMH study. The abbreviated ATIS (see Appendix) includes six items which are all scored on a five-point scale (0 = Never, 1 = Rarely, 2 = Sometimes, 3 = Often, 4 = Always). The six-item scale produced an internal consistency index of α = 0.63.

#### Cultural engagement

Cultural engagement was measured as a composite score of three questions (scores on a five-point scale) from the broader Social and Emotional Wellbeing and needs/service access questionnaire. Items were summed to create total scores ranging from 0 to 12 with higher scores indicating greater cultural engagement. The three items are: 1) How often do you participate in Aboriginal and Torres Strait Island activities or events? 2) Do you feel connected to your homeland or traditional country? 3) Do you feel connected to your culture? Item 1 was sourced from the original seven item ATIS. The internal consistency of the three item scale is α = .675.

### Data analysis

Descriptive statistics were employed to ascertain the mean and range of the abbreviated ATIS scale and the composite cultural engagement measure. A univariate ANOVA analysis was then conducted to determine if cultural identity predicted cultural engagement. Next, median splits for participant total scores on both measures were performed in order to divide the sample into high and low ‘identification’ and ‘engagement’ groups. A series of logistic regression analyses were then performed with a sample of released prisoners to determine whether cultural identity and cultural engagement alone predicted violent recidivism. Further logistic regression analyses were performed, this time to ascertain if cultural engagement predicted violent recidivism differently by strength of cultural identity. Finally, a survival analysis was conducted to determine differences in time to re-offence by the level of cultural engagement. Log-rank tests were used to identify group differences.

## Results

### Descriptive analyses

The mean total score on the abbreviated ATIS Scores was 21.08 (*SD* = 3.43) and ranged from 11 to 24. The median score was 22. For the cultural engagement measure, the mean score was 9.54 (SD = 2.60) and the range was 1 to 12. The median score was 10.

### Relationship between identity and engagement

A linear regression was conducted to determine if cultural identity predicted cultural engagement. Results found that cultural identity explained 35% of the variance [*F*(1117) = 65.10, *p* < .001] and significantly predicted cultural engagement [β = .60, *t*(117) = 8.07, *p* < .001]. Similarly, cultural engagement predicted cultural identity [β = .80, *t*(117) = 8.07, *p* < .001] explaining 35% of the variance [*F*(1117) = 65.10, *p* < .001]. Both cultural identity and cultural engagement were significantly correlated [*r* = .60, *p* < .01].

### Predictors of violent re-offending

The outcome sample was reduced to 84 as 33 participants had either not been released from custody during the follow-up period or they had not given researchers permission to access their official criminal histories. Four-2 % (*N* = 35) of the sample were charged with a violent re-offence during the follow-up period. Logistic regression analyses were conducted to determine if cultural identity and cultural engagement independently predicted violent recidivism over a 2 year period (see Table 1). Cultural engagement significantly predicted non-re-offense. This relationship remained after controlling for age [B(*SE*) = −.23(.01), Wald = 5.70, *Exp*(B) = .79, *p* = .02]. No meaningful relationship between identity and violent re-offence was discovered. Furthermore, the interaction between cultural engagement and cultural identity in the prediction of violence was not significant.

**Table 1:** Predictors of recidivism by engagement and identity

	B (SE)	Wald	*Exp*(B)	*p*
Cultural Engagement	−.226 (.09)	5.94	.80	.02
Cultural Identity	−.04 (.07)	0.27	.97	.60

*N* = 84

### Cultural engagement and recidivism by level of identity status

As cultural identity significantly predicted cultural engagement and cultural engagement predicted violent offending, the relationship between identity, engagement and violent offending was then explored. A median cut-off point of 22 was employed for the entire cohort to separate high and low Indigenous identification. Low identifiers (*N* = 29, 34.5% for the post-release cohort) included participants scoring 22 and below with high identifiers (*N* = 55, 65.5%) scoring between 23 and 24. No significant differences in age [*t*(82) = −.81, *p* = .31], previous number of times in prison [*t*(81) = −1.16, *p* = .87] or geographical region living within prior to custody (city/town/remote/other) [χ^2^(3) = 1.78, *p* = .62], were identified by level of identity.

Logistic regression analyses were then conducted to ascertain if cultural engagement predicted violent recidivism over a 2 year period across levels of cultural identity (see Table 2). Cultural engagement significantly predicted non-re-offense for high identifiers only. No significant relationship was found for low identifiers.

**Table 2:** Cultural engagement and violent recidivism by Indigenous identity status

	B (*SE*)	Wald	*Exp*(B)	*p*
High Identifiers (*N* = 55)	−.39	5.12	0.68	.02
Low Identifiers (*N* = 29)	−.33	3.30	0.72	.07

*N* = 84. High identifiers obtained scores >22 on the abbreviated ATIS. Low identifiers obtained scores ≤22

### Cultural engagement and time at-risk

A survival analysis was performed to determine differences in time to re-offence by degree of cultural engagement.

A median cut-off point of 10 was employed to separate high and low cultural engagement. Participants with Low cultural engagement (*N* = 46, 54.8%) received scores from 1 to 10 and those with high cultural engagement (*N* = 38, 45.2%) received scores above 10.

**Fig. 1:**
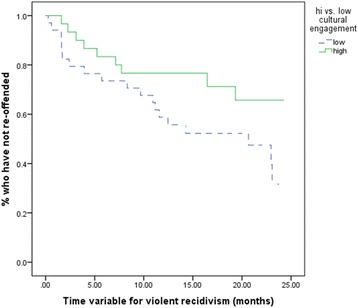
Time to re-offence by degree of cultural engagement

Figure 1 suggests that participants who had high levels of cultural engagement took longer to violently re-offend. The mean time to re-offence for participants who had low levels of cultural engagement was 15.28 (*SE* = 1.61) months while for participants with high levels of cultural engagement, time to re-offence was 18.93 (*SE* = 1.55) months, although this difference did not reach statistical significance [χ^2^(1) = 3.30, *p* = .07].

A second analysis was conducted to identify differences in time at-risk by strength of identity. The mean times to re-offence were almost equal across groups (Strong identity: 16.98 months; Weak identity: 16.93).

## Discussion

This study explored the relationship between cultural identity, cultural engagement and violent recidivism for a sample of Indigenous Australian people in custody. Findings indicate that cultural engagement contributes to criminal desistance denoting culture as an important part of institutional care for Indigenous Australians.

In the study, a stronger cultural identity predicted higher levels of cultural engagement. This means that conjointly having pride in one’s Indigeneity, possessing knowledge about one’s tribal background and ascribing a level of personal significance to cultural knowledge engendered greater engagement. Similarly, cultural engagement significantly predicted cultural identity. Both constructs were meaningfully correlated. This is unsurprising given that participation in cultural activities and developing a sense of connection to a cultural group are key aspects of cultural identity development and maintenance. For Dudgeon and Walker, ‘the notion of being Aboriginal – of being connected to family, kin, and community – continues to inform cultural identity’ [31]. For the custodial sample, identity and cultural engagement generally appear to fluctuate concurrently.

For participants who were released from custody during the study follow-up period, cultural engagement was significantly associated with non-recidivism. The observed protective impact of cultural engagement is a novel finding in a correctional context. Prior literature has shown that cultural attachment buffers against alcohol abuse [32] and suicidal behaviors [15, 33] in Native American populations, though participations are generally non-offenders and behavioral outcomes are not necessarily official law breaking activities. The negative relationship between cultural engagement and violent recidivism in this study may be explained through notions of enhanced self-assurance, self-esteem, life purpose, and social support which are often designated as consequences of cultural engagement/attachment [17]. The cultivation of life balance and support networks through cultural engagement may have also allowed for improved engagement in other pre-post custodial therapeutic initiatives. The concept of ‘culture as cure’ may have also provided culturally engaged participants with an array of cultural principles, customs, obligations and traditions to adhere to, conferring structure, life meaning and direction. The importance of cultural connectivity among Aboriginal men has been previously recognized in Australian custodial settings [34].

In contrast, cultural identity alone did not have a meaningful relationship with recidivism. However, whereas identity alone did not buffer recidivism directly, it may have had an indirect influence given its relationship with cultural engagement. It is plausible that a strong Indigenous identity enables greater cultural engagement which in turn lowers recidivism. Prison settings may engender identity salience as they are often environmentally partitioned along ethno-cultural lines. Alternatively, cultural engagement in custodial settings may have prompted both a stronger cultural identity and a lower likelihood violent recidivism. No significant interaction between cultural identity and cultural engagement was discovered in the prediction of violence. However sub-group analyses were warranted given that cultural identity significantly predicted cultural engagement. A median split across identity enabled further enquiry into this relationship. For participants with a strong Indigenous identity, cultural engagement in custody was significantly associated with non-recidivism. The effect was not significant for participants with a weak Indigenous identity although the result appeared to trend in a similar direction to participants with a strong identity. This reinforces earlier findings from this study that underscore the importance of cultural engagement/connection in reducing recidivism. The combination of a strong Indigenous identity and engagement/connection with culture influences the likelihood of violent recidivism. For participants with a weaker cultural identity, engagement with culture may not have had a commensurate impact on future offending for several reasons. Despite ‘engaging’ with culture in custody, weaker identifiers may not have had the cultural efficacy or the perceived resources or cultural knowledge to express cultural behaviors necessary for establishing a meaningful connection [35]. A recent NATSISS survey discovered that almost 5 out of 10 Aboriginal people in non-remote areas did not identify with a tribal or clan grouping [36]. It may be the case that the level of ‘cultural scaffolding’ that comes with a strong identity is necessary before a ‘connection’ eventuates beyond mere symbolism. Meaningfully engaging and/or connecting with Indigenous culture may have less ‘life impact’ if one knows little about their own Indigenous heritage or affords minimal importance to possessing Indigenous knowledge. Moreover, Berry contends that there are situations where ‘an individual may be induced to behave superficially as an Aboriginal person without the presence of the underlying identity’ [1].

Neither a strong identity nor greater cultural engagement significantly reduced time without recidivism, post-release. However, a trend towards significance was identified for participants who self-reported greater cultural engagement. It is possible that this effect may have reached significance specifically for participants who possessed a strong cultural identity. However, this outcome was not achievable, possibly due to small sample sizes.

### Limitations

The study has several limitations. First, Aboriginal and Torres Strait Islanders are heterogeneous peoples. Individuals may vary widely in how they identify culturally. For some, culture may be about speaking a traditional language, connection to the land or adherence to customary law. For others, attachment may be purely nominal. Several participants in the cohort may have harbored multiple identities without ascribing preference to their Indigenous identity. Nonetheless, the questions utilized to ascertain identity and engagement are in line with previous research and allow for the individual to determine ‘connection’ or ‘importance assigned to knowledge’ without specification. The median value used to partition both high and low identifiers and engagers were relatively high. This meant that for the cohort generally, possessing a strong identity and cultural engagement were common. As such, several participants in the weak identity or lower cultural engagement categories were in fact more likely to be ‘moderate’ adherents or partakers. Nonetheless, significant differences were still identified between groups. This suggests that a lower cut-off score may have produced larger group differences. Finally, caution is advised when interpreting the results as additional variables that were not controlled for could perhaps have influenced the findings. Repeat offending is often preceded by a concert of unmet needs, trauma histories and adverse circumstances. Participant age and environmental surroundings prior to custody, however, were commensurate across identity groups.

## Conclusion

The findings of the study emphasize a greater need for correctional institutions to accommodate Indigenous cultural considerations. Although Indigenous cultural activities are available, they are often subject to client inaccessibility, irregularity, understaffing, underfunding and the vagaries of institutional decision-making [37]. There is a clear need for both cultural strengthening services that help cultivate a strong Indigenous identity and regular opportunities to participate in cultural activities [38]. This may include reliable access to elders, Aboriginal liaison/wellbeing officers, and designated cultural areas in custody [39]. Prisons must also undergo independent cultural safety audits to ensure that their practices, programming and staff credentials and expectations are not in any way diminishing a client’s capacity to develop and express their Indigenous identity. This must extend beyond custodial environments to include throughcare arrangements.

These study findings do not imply that a strong identity and cultural attachment will definitely reduce violent offending. However, there is clearly a relationship here, in particular that cultural engagement can reduce recidivism. Cultural considerations must be one component of holistic care – either as a complimentary part of rehabilitation or perhaps forming the basis for rehabilitation depending on client cultural needs. A recent review into the Northern Territory correctional services recommends that for young Aboriginal people in custody, healing time, spirituality and connection to culture should transpire prior to therapeutically addressing offending-specific behaviors [40]. A client with a strong identity who is engaged in cultural activities may develop the internal motivation to participate meaningfully in other programs and/or desist from criminal activity.

This study identified that cultural engagement in custodial settings was associated with a lower likelihood of violent re-offence. Furthermore, a strong relationship was found between cultural identity and cultural engagement. These findings provide evidence for the utility and importance of cultural initiatives in custody for Indigenous people.
